# Physical Activity Is Associated With Better Executive Function in University Students

**DOI:** 10.3389/fnhum.2020.00011

**Published:** 2020-02-18

**Authors:** Diana Salas-Gomez, Mario Fernandez-Gorgojo, Ana Pozueta, Isabel Diaz-Ceballos, Maider Lamarain, Carmen Perez, Martha Kazimierczak, Pascual Sanchez-Juan

**Affiliations:** ^1^Gimbernat-Cantabria Research Unit (SUIGC), University Schools Gimbernat-Cantabria, Attached to the University of Cantabria, Torrelavega, Spain; ^2^University Schools Gimbernat-Cantabria, Attached to the University of Cantabria, Torrelavega, Spain; ^3^Service of Neurology, University Hospital “Marqués de Valdecilla”, University of Cantabria (UC), CIBERNED, IDIVAL, Santander, Spain

**Keywords:** neuropsychological tests, physical activity, executive function, sex factors, women, young adult

## Abstract

**Introduction**: In recent years, the study of the benefits that physical exercise has on brain health has acquired special relevance. In order to implement exercise as an intervention to protect the brain, it is important to have a more clear idea of its effect in the young population. However, few studies have been carried out on these ages.

**Objective**: The main objective of our study was to evaluate the association between physical activity (PA) with memory and executive function, in university students, analyzing the modulatory effect of sex.

**Methodology**: We collected socio-demographic and life habit information, as well as data on the PA that was carried out during the previous week using the international PAquestionnaire short version (IPAQ-SF) questionnaire in 206 university students (mean age 19.55 ± 2.39; 67.5% women). Memory and executive function were assessed using a comprehensive battery of validate cognitive tests. Univariate and multivariate analyses were performed to correlate PA with cognitive tests scores and to evaluate the potential synergistic role of sex.

**Results**: The main finding was that the total amount of PA correlated positively with several tests that evaluated aspects of executive function, specifically Stroop Colors (Pearson’s *r* = 0.17; *p* = 0.01) and the Stroop Test Color–Word (Pearson’s *r* = 0.15. *p* = 0.03). These results were adjusted by a large number of possible confounders and modifying variables in a multivariate analysis, like age, sex, academic record, day of the week, and time at which the test was performed. Additionally, we found out that sex had a synergistic effect with PA on the executive test Trail making test-A (TMTA), and in women, this association was stronger than in men. The more PA women reported, the better they performed, that is to say that they took less time to finalize the TMT-A (interaction term between PA and sex: *b* = −0.0009; *p* = 0.014).

**Conclusion**: Our study adds evidence of the benefit of PA in cognition in the young population, specifically in the executive inhibitory control, and more significantly in women.

## Introduction

In recent years, the study of the effects of physical activity (PA) on brain health and the improvement of cognitive function has acquired special attention. This has been mainly driven by findings from several studies reporting the association between PA and active lifestyle and a decrease in dementia risk and cognitive improvement at old age (Larson et al., 2006; Wang and van Praag, 2012; Bherer et al., 2013; Prakash et al., 2015; Duzel et al., 2016; Santos-Lozano et al., 2016; Engeroff et al., 2018). It has also been considered as a potential strategy to improve academic performance, cognitive abilities, and intellectual function in children (Tomporowski et al., 2008), although the evidence for this is limited (Li et al., 2017).

There have been an increasing number of randomized clinical trials addressing the effect of PA on cognition in different age groups (Best, 2010; Liu-Ambrose et al., 2010; Nouchi et al., 2014; Iuliano et al., 2015; Álvarez-Bueno et al., 2017). Several meta-analyses supported the causality of this association, showing a low-to-moderate effect size on the improvement of cognitive aspects, especially executive function, after aerobic exercise sessions (Chang et al., 2012; Verburgh et al., 2014; Ludyga et al., 2016; de Greeff et al., 2018; Loprinzi et al., 2019) or high intensity and frequency (Wang et al., 2019).

From a basic research perspective, it has been reported that regular PA has a direct effect at the neuronal level, enhancing synapses and vascularization (Wang and van Praag, 2012; Erickson et al., 2015). Animal studies support the hypothesis that brain-derived neurotrophic factor (BDNF) would play a key role in this process (Wang and Holsinger, 2018). In the young adult, who is at a critical stage in perfecting neuronal pathways and strengthening synapses, PA may become especially important in the development of brain functions (Tomporowski et al., 2008). However, the mechanisms by which cognitive abilities improve in physically active individuals are not fully understood (Lautenschlager et al., 2008).

Although pooled analyses are consistent with an association, there are relevant discrepancies between authors, and cognitive function improvements after PA interventions are not conclusive in all trials (Colcombe and Kramer, 2003; Hillman et al., 2006; Smith et al., 2010; Gates et al., 2013; Öhman et al., 2014; Iuliano et al., 2015; Rezab, 2015; Cox et al., 2016; Ludyga et al., 2016; Barha et al., 2017). Differences may be due to issues such as the type of PA intervention, the targeted populations, the neurocognitive tests selected as main outputs, and the time period of the trial (Martín-Martínez et al., 2015; Martínez et al., 2017).

There are relevant aspects that need to be further elucidated. Most of the studies assessed PA either in the middle and later stages of adult life or in childhood–adolescence. There is currently insufficient information on the influence of PA on young adults (Guiney and Machado, 2013; Verburgh et al., 2014; Cox et al., 2016; Engeroff et al., 2018). When assessing the effect of acute PA, the benefit on cognitive functions appears to be clear in children and adolescents. However, when chronic PA is evaluated in different populations, the benefit is not yet entirely clear. In this sense, it is particularly important to carry out well-designed longitudinal studies, since today, we have a very sedentary young population, and this may influence the development of executive functions in the long term (Verburgh et al., 2014).

The cognitive pattern associated with PA is not fully established, as comprehensive assessments systematically exploring memory and executive domains are not frequently published, and many of the studies report on specific cognitive tests only.

There is some evidence that biological sex has a differential influence on the type of memory. These differences, moderated by psychological factors and physiological parameters, could be modified in response to PA (Loprinzi and Frith, 2018). Along these lines, two meta-analyses conducted in 2003 and 2017 concluded that studies that included more women tended to show greater cognitive benefits associated with PA than studies with fewer women (Colcombe and Kramer, 2003; Barha et al., 2017). A randomized, controlled clinical trial evaluating the effect of acute PA on memory found that young women performed episodic memory tasks better than men (Johnson and Loprinzi, 2019). In addition to memory, studies evaluating the effect of endurance PA showed in elderly women a clear benefit on executive functions (Liu-Ambrose et al., 2010). Although in this previous study there was no male control group, we hypothesize that PA has a positive effect on executive function cognitive functions, and this effect could be more relevant in women.

The main objective of our study was to evaluate the association between PA with memory and executive function, in university students, analyzing the modulatory effect of sex.

## Materials and Methods

### Design

This is a cross sectional study to assess the association between reported PA and cognitive performance determined by a comprehensive battery of neurocognitive tests (von Elm et al., 2008).

### Participants

The study included all university students enrolled in the academic year 2013–2014 at University Schools Gimbernat—Cantabria, Attached to the University of Cantabria. Exclusion criteria were a history of severe cranioencephalic trauma, neurological diseases, dyslexia, color blindness, difficulties with the Spanish language, and sensory deficits.

The study was reviewed and approved by our institutional review board (Cantabria Research Ethics Committee ref. 2012.152), and we followed the ethical principles of the Declaration of Helsinki (World Medical Association, 2020). All participants signed an informed consent before entering the study. At study baseline, all participants were over 18 years of age except for eight individuals aged 17. They were all first year students that turned 18 during the academic course. In agreement with our review board, the eight under-18 participants signed the written informed consent document during the study period once they turned 18 years.

### Materials and Procedure

We interviewed all participants in two individual sessions lasting for 30 min. First, participants filled out the international PA questionnaire short version (IPAQ-SF; Rangul et al., 2008). The consumption of alcohol and other drugs, like cannabis, and sociodemographic information were also registered. In the second part, we administered to all participants a battery of neurocognitive tests validated for the study of young population and aimed at assessing memory and executive functions (Peña-Casanova et al., 2012). The assessors were given prior training in order to administer and evaluate correctly the cognitive tests in a standardized way. A logical memory test was given (WMS-III; Sueiro and Pereña, 2004; Aguirre-Acevedo et al., 2019), together with the CERAD word list (Morris et al., 1989; Mirra et al., 1991) to study episodic verbal memory; the Rey–Osterrieth complex figure test (ROCF), copy and recall (Tulsky et al., 2003) to test for constructional apraxia and differed visual memory; the digit span test WAIS-III (Sueiro and Pereña, 2004; Aguirre-Acevedo et al., 2019) to test working memory, attention span, and concentration; the standardized version of the color word test (STROOP) to check capacity to inhibit automatic response and the Trail making tests (TMT) A and B to evaluate visual–motor speed (part A) and attention and mental flexibility (part B; Golden, 2001; Golden and Freshwater, 2002).

### Statistical Analysis

To quantify the PA based on the metabolic equivalents (MET), following the criteria established by the IPAQ, we proceeded by calculating the total METs corresponding to a week (Ara, 2005). Brisk walking was equal to 3.3 METs, moderate and vigorous PA to 4 and 8 METs, respectively. Thus, the quantification of the PA was done using the following formula:

Total PA = brisk walking (3.3 METs ×minutes ×amount of weekdays) + moderate PA (4 METs × minutes × amount of weekdays) + vigorous PA (8 METs × minutes × amount of weekdays).

Pearson’s test was used to evaluate the correlation between the total METs of PA and the results of the cognitive tests. In addition, the participants were categorized according to their alcohol habits, dividing them into binge drinkers (BD) or non-binge drinkers (non-BD). A multivariant analysis was conducted through ANCOVA containing possible confounders or modifying variables of the effect, including the PA, age, sex, academic record, day of the week and time at which the test was performed, the person carrying out the examination, and whether they were or not categorized as BD. We assessed the interaction between sex and PA for each of the neuropsychological tests using ANCOVA. Additionally, the possible interaction between PA and sex was evaluated by a simple moderation analysis with the process package for SPSS (Bolin, 2014; Hayes and Little, 2018).

All statistical analyses were carried out using SPSS 19.0 (Statistical Product and Service Solutions IBM SPSS Statistics 19.0 2010).

## Results

The final sample included 206 individuals, with a mean age of 19.55 ± 2.39 years, of whom 67.5% were women. Only two foreign students were excluded due to their lack of understanding of the Spanish language.

The students performed on average 1.5–2 days of vigorous PA (e.g., running, swimming, or biking), with a mean of 37.5–57.9 min per day. Days per week spent on moderate PA were 1.4–1.7, employing 37.5–45.2 min per day on average. Table 1 shows the most relevant sociodemographic characteristics and PA habits.

**Table 1:** Sociodemographic characteristics and international physical activity questionnaire (IPAQ) results.

	Total (*N* = 206)
Years of age (mean ± SD)	19.6 ± 2.4
Women (%)	67
Academic record 0/10 (mean ± SD)	6.3 ± 1.4
Days per week of vigorous activity (mean ± SD)	1.5 ± 2
Minutes per day of vigorous activity (mean ± SD)	37.5 ± 57.9
MET of vigorous activity (mean ± SD)	900.5 ± 1685.1
Days per week of moderate activity (mean ± SD)	1.4 ± 1.7
Minutes per day of moderate activity (mean ± SD)	37.5 ± 45.2
MET of moderate activity (mean ± SD)	335.2 ± 512.9
Days in which they walked >10 (mean ± SD)	5.4 ± 2.1
MET walking (mean ± SD)	811.5 ± 986.5
MET TOTAL (mean ± SD)	2,043.3 ± 2,111.3

*Abbreviation: MET, metabolic equivalent*.

**Figure 1:**
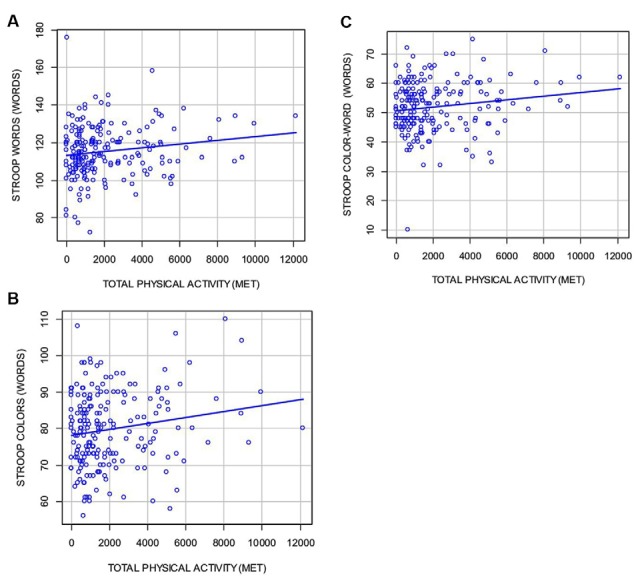
Scatter plots showing Stroop test results (**A** = words, **B** = colors, and **C** = color-word) and their correlation with the total amount of physical activity (PA) in metabolic equivalent (METs).

Table 2 and Figure 1 depict the students’ neuropsychological test results and their correlation with the total amount of METs of PA carried out weekly. A statistically significant positive correlation was found for Stroop Test Words (Pearson’s *r* = 0.157; *p* = 0.024), Stroop Test Colors (Pearson’s *r* = 0.17; *p* = 0.01), and the Stroop Test Color–Word (Pearson’s *r* = 0.15. *p* = 0.03). That is to say, the more PA the students performed, the more items were correctly named in the three subtests.

**Table 2:** Neuropsychological test results and their correlation with the total amount of physical activity in METs.

Neuropsychological test	Pearson’s *r* correlation with total physical activity	*p*-value	*p*-value^^^
WMS-III Logical memory word	−0.09	0.18	0.23
ROCF copy score	0.09	0.23	0.68
ROCF delayed recall	0.10	0.17	0.33
CERAD word list memory	0.04	0.55	0.99
CERAD word list recall	0.06	0.44	0.42
Digit span forward	0.06	0.36	0.22
Digit span backward	−0.03	0.63	0.99
Stroop Test Words	0.16*	0.02*	0.13
Stroop Test Colors	0.17*	0.02*	0.01*
Stroop Color–Word	0.15*	0.03*	0.03*
WMS logical memory delayed recall	0.01	0.88	0.91
TMT-A	−0.13	0.07	0.34
TMT-B	−0.12	0.08	0.29

Note: *p < 0.05, ^p-values were adjusted by age, sex, academic record, day of the week, time at which the test was performed, the person carrying out the examination, and binge drinking pattern. Abbreviations: TMT-A, Trail making test A; TMT-B, Trail making test B.

We used ANCOVA adjusting for age, sex, academic record, BD, and other possible modifying variables such as the week of the day and the time of the day the tests were conducted. The Stroop Test Colors, and the Stroop Test Color–Word remained statistically associated to PA. TMT-A and TMT-B were borderline, which significantly correlated at the univariate analysis TMT-A (Pearson’s *r* = −0.13; *p* = 0.07 and Pearson’s *r* = −0.12; *p* = 0.08), which means that individuals with higher levels of PA tended to perform these tests better, that is, in fewer seconds. However, after adjustment for covariates, these results did not reach statistical significance. No other cognitive test was significantly correlated with PA.

**Figure 2:**
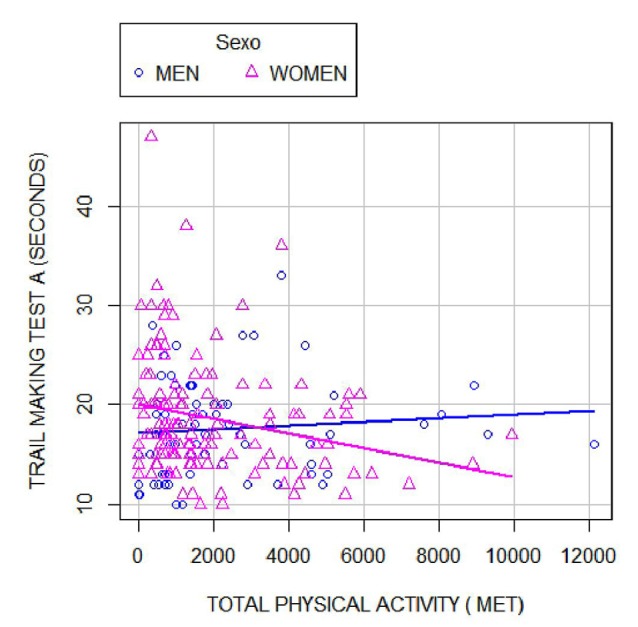
Scatter plot showing Trail making test A results and their correlation with the total amount of METs of PA stratified by sex.

Last, we evaluated the effect of PA on the neuropsychological tests measuring the executive function with regard to the sex. Table 3 and Figure 2 show that in the stratified analysis, there was a negative correlation with the TMT-A test, which was only statistically significant in women (Pearson’s *r* = −0.26; *p* = 0.01). Therefore, the more PA women performed, the better they did the task, spending less time to accomplish it. Finally, we assessed the interaction between sex and PA for each of the neuropsychological tests using ANCOVA. TMT-A was the only one in which the interaction term sex and total PA was statistically significant (*p* = 0.035). To gain a better understanding of this interaction, we carried out a simple moderation analysis. These results are shown in Table 4 and again reflects that in our study, sex played a significant modulating effect on the influence of PA on the executive function test TMT-A (*b* = −0.0009; *p* = 0.014).

**Table 3:** Correlations between the total METs of physical activity and the neuropsychological tests stratified by sex.

Neuropsychological	Men (*N* = 67)		Women (*N* = 139) test	
	Pearson’s *r*	*p*-value	Pearson’s *r*	*p*-value^^^
Stroop Test Words	0.22	0.16	0.09	0.54
Stroop Test Colors	0.27	0.06	0.11	0.40
Stroop Test Color–Word	0.25	0.08	0.09	0.60
TMT-A	0.09	0.88	−0.26*	0.01*
TMT-B	0.03	0.99	−0.20	0.04

Note: *p < 0.05, ^^^Bonferroni-adjusted p-value.

**Table 4:** Moderating effect of sex on the relationship between the physical activity and TMT-A.

Dependent variable	Predictor	b	se	*t*	*p*-value
**1. Interaction effect of SEX on the TMT-A**					
TMT-A	Total physical activity	−0.0003	0.0002	−1.4660	0.1442
	Sex	2.9378	1.1343	2.5899	0.0103
	Total physical activity*sex	−0.0009*	0.0004	−2.4905	0.0136*

**Sex**	**Effect**	**Se**	***t***	***p*-value**	**95% CI**
**2. Conditional effects of the physical activity at sex type**					
Male	0.0002	0.0003	0.6908	0.4905	−0.0004; −0.0007
Women	−0.0007	0.0002	−2.9484	0.0036	−0.0012; −0.0002

*p < 0.05.

## Discussion

In our analysis, we observed that there was a statistically significant positive correlation between PA and all components of the Stroop test (words, colors, and color–word). After multivariate analysis, colors and color–word subtests remained significant, adjusted by the main covariates and potential confounding factors.

The Stroop test consists of three subtests: in the first one, the subject must read the words “red,” “green,” and “blue” printed in black ink and randomly arranged in columns; in the second subtest, he must name the color of the ink on which the symbols “XXX” are printed, arranged in columns; and on the third subtest, he must name the color of the ink on which the words “red,” “green,” and “blue” are printed, but not read the word. The score of each sheet consists of the number of items read in 45 s. Stroop test evaluates key components of the executive function, chiefly the ability to inhibit stimuli that trigger automatic responses, cognitive control capacity, and mind flexibility, thus, our main finding suggests that the increased PA may improve these cognitive abilities in young adults since the more PA, the greater the number of items the participants were able to read.

Similar findings were shown in a study with preadolescents (between 7 and 12 years old; Buck et al., 2008). In this population, age was reported as a significant modulator of the effect of PA on the three Stroop tests, that is, the older the participants, the larger was the effect size of PA on interference control. Our multivariate analysis showed that age was not an effect modulator for our population; however, our volunteers were older, and their age range was narrower. A different degree of prefrontal cortex (PFC) maturation, which is related to the development of inhibitory control, between both populations might explain the diverse effects observed (Dahl, 2004). It was postulated that aerobic exercise could have a greater impact on those individuals in which the executive function is still developing (Ludyga et al., 2016). On the other hand, in an elderly population (on average 79 years), similar results were also shown by other authors who found that the amount of PA explained a small, but significant part of the variance in Stroop color scores and interference (Bixby et al., 2007).

There are a few other observational studies evaluating the effect of a physically active lifestyle on executive function. Hillman et al. (2006) reported a faster reaction time associated with PA in subjects between 15 and 71; this result was independent of age. On the other hand, a recent study, carried out in university students, of a similar age range as our population, that evaluated the relationship between PA, reported by the IPAQ (long version), and executive function, found no association (Ho et al., 2018). Despite a similar design and target population, there are several methodological differences. First, the instrument used to evaluate the executive function differed between studies. Ho et al. (2018) used the flanker task—a task related to attention and inhibition that has been shown to activate similar brain regions in functional MRI as the Stroop test, such as the anterior cingular cortex (APP) and left prefrontal cortex (lPFC; Fan et al., 2003). Despite the similarity of the two neurocognitive tests, a study reported that the time needed to resolve the response conflict in Stroop’s task did not predict the time needed to resolve the response conflict in the flanker task, and therefore, the interference scores between the two tasks were not correlated. That led the authors to postulate that, although both tasks require the use of the same brain regions, the Stroop test is more demanding of frontal executive resources since this test involves a verbal output and implies a more diverse set of stimuli (Stins et al., 2005). In addition, these discordances may also be due to the different versions of the IPAQ that were used. While they opted for the long version, for our study, we chose the short version (IPAQ-SF). The IPAQ is an instrument that has been validated in a multitude of countries like Spain (Rangul et al., 2008; Román Viñas et al., 2013), and it has been widely used in diverse populations in its long as well as short version (Craig et al., 2003; Rodríguez-Muñoz et al., 2017; Rubio et al., 2017). A recent study carried out in Spanish university students aimed to validate the IPAQ-SF with an objective measurement of the PA through accelerometers, concluding that this questionnaire was a reliable tool to assess PA in the same population as our study (Rodríguez-Muñoz et al., 2017).

The second objective of this study was to assess whether sex modulates the effect of PA on cognitive functions. TMT-A was the only test in which the interaction term of sex and PA was statistically significant. TMT-A evaluates the attentional function, perceptual–motor speed capability, visual tracking skills, visoconceptual exploration, and visomotor exploration. Thus, our finding suggests that the increased PA may be related to these cognitive abilities in women.

This finding was consistently reported in the literature. In several clinical trials assessing the role of PA on mild cognitive impairment, greater benefits of aerobic training and resistance on cognition in women, specifically on executive function, were observed (van Uffelen et al., 2008; Baker et al., 2010; Nagamatsu et al., 2012). Similarly, a recent meta-analysis of clinical trials conducted in healthy volunteers, older than 45 years, also showed a greater improvement in executive function in those studies with a higher proportion of women. The largest improvement was obtained in those studies with aerobic interventions, resistance, or multimodal training performed for at least 2 months and at least once a week (Barha et al., 2017). Despite these findings, there is no clear explanation why in women, the effect of PA is greater than in men. Our study adds further evidence, reporting, as far as we know, for the first time, a statistically significant interaction term between sex and PA in a multivariate model.

No association was found with PA and any of the other neurocognitive tests carried out in our study. Functions such as episodic verbal memory, constructive apraxia, delayed visual memory, and working memory did not change significantly according to the levels of PA. There is little research on the influence of PA on these functions in young people. As a possible explanation, it has been postulated that brain at this age achieves its maximum development in areas related to these functions so that it would be difficult to obtain a further improvement of these cognitive abilities (Salthouse and Davis, 2006; Rezab, 2015). We hypothesize that the specificity of the effect of PA on executive control could be related to the fact that areas of the brain, mainly the dorsolateral prefrontal cortex (dPFC), associated with this function are still developing in this age population. These same areas are more vulnerable to toxins such as alcohol in this period of life (Gill, 2002; White and Swartzwelder, 2004; Casey and Jones, 2010; Salas-Gomez et al., 2016). In a previous publication, we described that binge drinking was associated with worse executive function in this same population, and this effect was stronger in women, which is exactly the reverse pattern of what we find with PA (Salas-Gomez et al., 2016).

The IPAQ questionnaire presents obvious limitations since it only refers to the PA carried out in the previous week. This could be problematic for a population of students, as factors like the academic calendar might influence the amount of PA performed in a given week.

Another limitation was in quantifying the amount of PA using indirect tools such as IPAQ. However, this questionnaire has been widely validated, and as mentioned above, a recent study proved its usefulness in Spanish university students (Rodríguez-Muñoz et al., 2017). On the other hand, our multivariate analysis allowed adjusting by the main covariates and potentially relevant confounders, like the day of the week and the time of the day when the assessment was performed, or the rater who performs the test. Due to our cross-sectional design, our results are potentially subject to bias. For example, reverse causality could be an alternative interpretation for our data. It has been reported that professional cyclists have a higher inhibitory control than amateurs and performed significantly better in the Stroop test, leading the authors to hypothesize that their superior ability would allow them to cope with higher levels of mental fatigue and, thus, become elite athletes (Martin et al., 2016). Causality between PA and improved executive function has been consistently tested in prospective clinical trials, so we consider this explanation unlikely.

## Conclusion

In conclusion, our study adds further evidence for the beneficial relationship between an indirect measurement of PA, through the internationally validated IPAQ-SF questionnaire, and cognition in young adults. Specifically, our findings suggest that the practice of PA might improve aspects of executive function such as the ability to inhibit stimuli that trigger automatic responses, cognitive control ability, and mental flexibility in university students. In addition, we found a synergistic effect between PA and sex, with a more intense association with females regarding attention function and perceptual–motor speed capacity.

## Data Availability Statement

The datasets generated for this study are available on request to the corresponding author.

## Ethics Statement

The studies involving human participants were reviewed and approved by Cantabria Research Ethics Committee. The patients/participants provided their written informed consent to participate in this study.

## Author Contributions

PS-J participated in the design of the study and the funding acquisition. He contributed to the formal analysis and methodology. He also participated in project administration, supervision, writing, review, and editing. DS-G participated in data curation, informal analysis, and in the investigation. She also contributed to the methodology, writing, and the original draft. MF-G participated in data curation and in the investigation. He also contributed to the methodology, writing, and the original draft. AP, ID-C, ML, and CP contributed to the investigation. All authors read and approved the final version of the manuscript, and agreed with the order of presentation of the authors.

## Conflict of Interest

The authors declare that the research was conducted in the absence of any commercial or financial relationships that could be construed as a potential conflict of interest.
